# Aqua­(2-hydr­oxy-5-sulfonatobenzoato-κ*O* ^1^)bis­(2-phenyl-1*H*-1,3,7,8-tetra­aza­cyclo­penta­[*l*]phenanthrene-κ^2^ *N* ^7^,*N* ^8^)zinc(II)

**DOI:** 10.1107/S1600536809039154

**Published:** 2009-10-03

**Authors:** Qiang Han, Xiang-Cheng Wang, Xiu-Ying Li, Guan-Xin Yao, Yong-Sheng Yan

**Affiliations:** a School of Material Science and Engineering, Jiangsu University, Zhenjiang 212013, People’s Republic of China; b School of Chemistry and Chemical Engineering, Jiangsu University, Zhenjiang 212013, People’s Republic of China

## Abstract

In the title compound, [Zn(C_7_H_4_O_6_S)(C_19_H_12_N_4_)_2_(H_2_O)], the Zn^II^ ion is coordinated by two *N*,*N*′-bidentate 2-phenyl-1*H*-1,3,7,8-tetra­azacyclo­penta­[*l*]phenanthrene ligands, one *O*-monodentate 5-sulfosalicylate dianion and a water mol­ecule. This results in a distorted *cis*-ZnO_2_N_4_ octa­hedral coordination geometry for the metal ion. In the crystal, mol­ecules are expanded into a three-dimensional supra­molecular motif *via* O—H⋯O, O—H⋯N and N—H⋯(O,S) hydrogen bonds. In addition, π–π stacking inter­actions between the aromatic rings of the polycyclic ligands consolidate the sturcture [shortest centroid–centroid distance = 3.501 (2) Å].

## Related literature

For related structures, see: Che *et al.* (2008 ▶); Li *et al.* (2009 ▶); Liu *et al.* (2009 ▶). For the synthesis of the ligand, see: Steck & Day (1943 ▶).
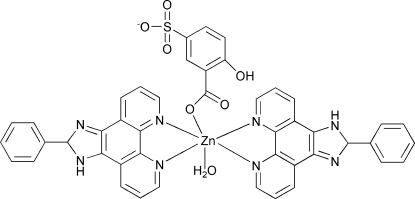


## Experimental

### 

#### Crystal data

[Zn(C_7_H_4_O_6_S)(C_19_H_12_N_4_)_2_(H_2_O)]
*M* *_r_* = 892.20
Monoclinic, 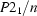

*a* = 8.3257 (8) Å
*b* = 25.926 (2) Å
*c* = 18.3271 (13) Å
β = 101.259 (8)°
*V* = 3879.8 (6) Å^3^
*Z* = 4
Cu *K*α radiation
μ = 1.94 mm^−1^
*T* = 292 K
0.27 × 0.26 × 0.23 mm

#### Data collection

Oxford Diffraction Gemini R Ultra diffractometer
Absorption correction: multi-scan (*CrysAlis RED*; Oxford Diffraction, 2006 ▶) *T*_min_ = 0.621, *T*_max_ = 0.640
15767 measured reflections
6808 independent reflections
4337 reflections with *I* > 2σ(*I*)
*R*_int_ = 0.055

#### Refinement

*R*[*F* ^2^ > 2σ(*F* ^2^)] = 0.049
*wR*(*F* ^2^) = 0.132
*S* = 0.97
6808 reflections
575 parameters
2 restraints
H atoms treated by a mixture of independent and constrained refinement
Δρ_max_ = 0.41 e Å^−3^
Δρ_min_ = −0.28 e Å^−3^

### 

Data collection: *CrysAlis CCD* (Oxford Diffraction, 2006 ▶); cell refinement: *CrysAlis CCD*; data reduction: *CrysAlis RED* (Oxford Diffraction, 2006 ▶); program(s) used to solve structure: *SHELXS97* (Sheldrick, 2008 ▶); program(s) used to refine structure: *SHELXL97* (Sheldrick, 2008 ▶); molecular graphics: *SHELXTL-Plus* (Sheldrick, 2008 ▶); software used to prepare material for publication: *SHELXL97*.

## Supplementary Material

Crystal structure: contains datablocks global, I. DOI: 10.1107/S1600536809039154/hb5120sup1.cif

Structure factors: contains datablocks I. DOI: 10.1107/S1600536809039154/hb5120Isup2.hkl

Additional supplementary materials:  crystallographic information; 3D view; checkCIF report

## Figures and Tables

**Table 1:** Selected geometric parameters (Å, °)

Zn—O1	2.080 (3)
Zn—O*W*1	2.196 (3)
Zn—N1	2.177 (3)
Zn—N2	2.184 (3)
Zn—N5	2.141 (3)
Zn—N6	2.120 (3)

**Table 2:** Hydrogen-bond geometry (Å, °)

*D*—H⋯*A*	*D*—H	H⋯*A*	*D*⋯*A*	*D*—H⋯*A*
O3—H3*C*⋯O2	0.82	1.82	2.547 (4)	147
O*W*1—H1*WA*⋯O2	0.86 (2)	1.90 (3)	2.712 (4)	157 (5)
O*W*1—H1*WB*⋯N7^i^	0.840 (19)	2.05 (2)	2.877 (4)	170 (4)
N4—H4*B*⋯O6^ii^	0.86 (4)	2.05 (4)	2.883 (4)	162 (4)
N4—H4*B*⋯S1^ii^	0.86 (4)	3.02 (4)	3.854 (4)	163 (3)
N8—H8*B*⋯O4^iii^	0.96 (5)	1.85 (5)	2.794 (5)	167 (5)
N8—H8*B*⋯S1^iii^	0.96 (5)	2.94 (5)	3.793 (4)	148 (4)

Symmetry codes: (i) 
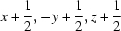
; (ii) 
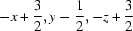
; (iii) 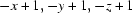.

**Fig. 1.**
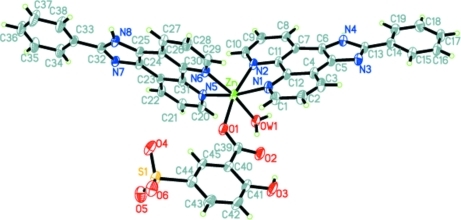
View of the local coordination of compound (I) with displacement ellipsoids drawn at the 30% probability level. (arbitrary spheres for the H atoms).

**Fig. 2.**
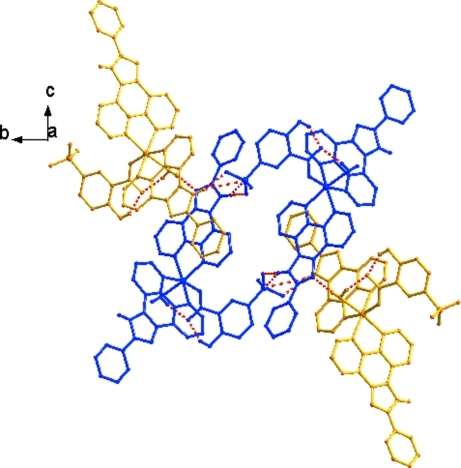
View of three-dimensional superamolecular structure of (I) built up via hydrogen bonds and π-π interactions. Most H atoms have been omitted.

## supplementary crystallographic information

### Comment

1,10-phenanthroline (phen) and its derivatives has been widely used to build supramolecular architectures owing to their excellent coordinating ability and large conjugated system (Che *et al.*, 2008; Li *et al.*, 2009). Whenas, building blocks derived from the appropriate modification of phen, such as 2-phenyl-1*H*-1,3,7,8-tetra-azacyclopenta[*l*]phenanthrene (*L*) have received considerably less attention (Liu *et al.*, 2009). Hereby, we have prepared the title compound, namely, [Zn(C_19_H_12_N_4_)_2_(C_7_H_4_O_6_S)H_2_O] or [Zn(*L*)_2_ (HSSA)H_2_O] (I), based on *L* and 5-sulfosalicylic acid (H_3_SSA) ligands.

In the compound (I), each Zn atom is six-coordinated by four N atoms from two *L* ligands, one O atom from a HSSA ligand and one water molecule (Fig. 1). The Zn—O distances range from 2.080 (3) Å to 2.196 (3) Å and the Zn—N lengths from 2.120 (3) to 2.184 (3) Å (Table 1). The N1, N5, N6, O1W atoms comprise the basal plane, while the O1 and N2 atoms occupy the axial position. In (I), –CO_2_H and –SO_3_H groups are deprotonated, but –OH groups are neutral. The carboxylate group of a HSSA ligand displays monodentate bridging coordination mode, whenas –OH and –SO_3_^-^ groups are uncoordinated.

The neighboring mononuclear Zn^II^ units interact by various hydrogen bonds, leading to a three-dimensional supramolecular structure (Fig. 2): (*a*) N—H···O or N—H···S hydrogen bonds between imidazole rings donors and the sulfonic groups of the HSSA ligands [N4···O6^i^: 2.883 (4) Å; N4···S1^i^: 3.854 (4) Å; N8···O4^ii^: 2.794 (5) Å; N8···S1^ii^: 3.793 (4) Å, symmetry code: (i) -*x* + 3/2, *y* - 1/2, -*z* + 3/2, (ii) -*x* + 1, -*y* + 1, -*z* + 1]. (*b*) O—H···O or O—H···N hydrogen bonds involving the coordinated water molecule OW1 and the O2, N7^ii^ atoms [O1W···O2: 2.712 (4) Å; O1W···N7^iii^: 2.877 (4) Å, symmetry code: (iii) *x* + 1/2, -*y* + 1/2, *z* + 1/2]. (*c*) Intramolecular O—H···O hydrogen bonds involving hydroxy oxygen atom of HSSA anion and carboxylate O2 atom [O3···O2: 2.547 (4) Å] (Table 2). In addition, π-π stacking interactions between *L* ligands further intensify the current architectures with a shortest stacking distance of 3.501 (2) Å.

### Experimental

The *L* ligand was synthesized according to the literature method (Steck & Day, 1943). A mixture of *L*, H_3_SSA, Zn(NO_3_)_2_ and water in the mole ratio 1:1:1:4000 was placed in a 25 ml Teflon-lined autoclave and heated for 4 d at 433 K under autogenous pressure. Upon cooling and opening the bomb, yellow blocks of (I) were obtained, which were washed with H_2_O and dried in air (62% yield based on Zn).

### Refinement

All H atoms on C atoms were positioned geometrically (C—H = 0.93 Å) and refined as riding, with *U*_iso_(H)= 1.2*U*_eq_(C). The hydrogen atoms of water molecules were located from difference Fourier maps and their positions and *U*_iso_ values were refined freely.

### Crystal data

**Table:**

[Zn(C_7_H_4_O_6_S)(C_19_H_12_N_4_)_2_(H_2_O)]	*F*(000) = 1832
*M**_r_* = 892.20	*D*_x_ = 1.527 Mg m^−^^3^*D*_m_ = 1.527 Mg m^−^^3^*D*_m_ measured by not measured
Monoclinic, *P*2_1_/*n*	Cu *K*α radiation, λ = 1.5418 Å
Hall symbol: -P 2yn	Cell parameters from 3619 reflections
*a* = 8.3257 (8) Å	θ = 4.9–67.0°
*b* = 25.926 (2) Å	µ = 1.94 mm^−^^1^
*c* = 18.3271 (13) Å	*T* = 292 K
β = 101.259 (8)°	Block, yellow
*V* = 3879.8 (6) Å^3^	0.27 × 0.26 × 0.23 mm
*Z* = 4	

### Data collection

**Table:**

Oxford Diffraction Gemini R Ultra diffractometer	6808 independent reflections
Radiation source: fine-focus sealed tube	4337 reflections with *I* > 2σ(*I*)
mirror	*R*_int_ = 0.055
Detector resolution: 10.2375 pixels mm^-1^	θ_max_ = 67.1°, θ_min_ = 4.9°
ω scans	*h* = −9→9
Absorption correction: multi-scan (Crys Alis RED; Oxford Diffraction, 2006)	*k* = −30→30
*T*_min_ = 0.621, *T*_max_ = 0.640	*l* = −21→19
15767 measured reflections	

### Refinement

**Table:**

Refinement on *F*^2^	Primary atom site location: structure-invariant direct methods
Least-squares matrix: full	Secondary atom site location: difference Fourier map
*R*[*F*^2^ > 2σ(*F*^2^)] = 0.049	Hydrogen site location: inferred from neighbouring sites
*wR*(*F*^2^) = 0.132	H atoms treated by a mixture of independent and constrained refinement
*S* = 0.97	*w* = 1/[σ^2^(*F*_o_^2^) + (0.0673*P*)^2^] where *P* = (*F*_o_^2^ + 2*F*_c_^2^)/3
6808 reflections	(Δ/σ)_max_ = 0.001
575 parameters	Δρ_max_ = 0.41 e Å^−^^3^
2 restraints	Δρ_min_ = −0.28 e Å^−^^3^

### Special details

**Table:**

Geometry. All e.s.d.'s (except the e.s.d. in the dihedral angle between two l.s. planes) are estimated using the full covariance matrix. The cell e.s.d.'s are taken into account individually in the estimation of e.s.d.'s in distances, angles and torsion angles; correlations between e.s.d.'s in cell parameters are only used when they are defined by crystal symmetry. An approximate (isotropic) treatment of cell e.s.d.'s is used for estimating e.s.d.'s involving l.s. planes.
Refinement. Refinement of *F*^2^ against ALL reflections. The weighted *R*-factor *wR* and goodness of fit *S* are based on *F*^2^, conventional *R*-factors *R* are based on *F*, with *F* set to zero for negative *F*^2^. The threshold expression of *F*^2^ > σ(*F*^2^) is used only for calculating *R*-factors(gt) *etc*. and is not relevant to the choice of reflections for refinement. *R*-factors based on *F*^2^ are statistically about twice as large as those based on *F*, and *R*- factors based on ALL data will be even larger.

### Fractional atomic coordinates and isotropic or equivalent isotropic displacement parameters (Å^2^)

**Table:**

	*x*	*y*	*z*	*U*_iso_*/*U*_eq_	
C1	0.8928 (5)	0.36143 (15)	0.8278 (2)	0.0515 (10)	
H1	0.8158	0.3878	0.8189	0.062*	
C2	1.0106 (6)	0.36325 (16)	0.8925 (2)	0.0577 (11)	
H2	1.0148	0.3908	0.9252	0.069*	
C3	1.1200 (5)	0.32385 (16)	0.9071 (2)	0.0542 (11)	
H3	1.1966	0.3235	0.9515	0.065*	
C4	1.1179 (5)	0.28381 (14)	0.85566 (19)	0.0413 (8)	
C5	1.2235 (5)	0.24000 (14)	0.86501 (19)	0.0440 (9)	
C6	1.2180 (5)	0.20368 (14)	0.80857 (19)	0.0419 (8)	
C7	1.1048 (4)	0.20682 (14)	0.73901 (18)	0.0399 (8)	
C8	1.0964 (5)	0.17231 (15)	0.6789 (2)	0.0465 (9)	
H8	1.1703	0.1451	0.6817	0.056*	
C9	0.9786 (5)	0.17933 (16)	0.6169 (2)	0.0517 (10)	
H9	0.9734	0.1577	0.5761	0.062*	
C10	0.8664 (5)	0.21915 (15)	0.6151 (2)	0.0496 (10)	
H10	0.7850	0.2230	0.5728	0.059*	
C11	0.9915 (5)	0.24714 (13)	0.73182 (18)	0.0399 (8)	
C12	0.9970 (5)	0.28631 (14)	0.79011 (18)	0.0408 (8)	
C13	1.3962 (5)	0.18131 (14)	0.90865 (19)	0.0433 (9)	
C14	1.5168 (5)	0.15014 (14)	0.9583 (2)	0.0446 (9)	
C15	1.5650 (5)	0.16544 (16)	1.0328 (2)	0.0517 (10)	
H15	1.5198	0.1949	1.0496	0.062*	
C16	1.6782 (5)	0.13724 (17)	1.0811 (2)	0.0580 (11)	
H16	1.7114	0.1482	1.1301	0.070*	
C17	1.7434 (6)	0.09257 (17)	1.0573 (2)	0.0600 (11)	
H17	1.8186	0.0731	1.0904	0.072*	
C18	1.6960 (5)	0.07698 (16)	0.9839 (2)	0.0585 (11)	
H18	1.7404	0.0472	0.9676	0.070*	
C19	1.5831 (5)	0.10548 (15)	0.9349 (2)	0.0499 (10)	
H19	1.5513	0.0946	0.8858	0.060*	
C20	0.4122 (5)	0.23843 (15)	0.5864 (2)	0.0499 (10)	
H20	0.4109	0.2184	0.6283	0.060*	
C21	0.3110 (5)	0.22443 (15)	0.5196 (2)	0.0515 (10)	
H21	0.2462	0.1950	0.5168	0.062*	
C22	0.3083 (5)	0.25462 (14)	0.4581 (2)	0.0474 (9)	
H22	0.2404	0.2461	0.4132	0.057*	
C23	0.4081 (4)	0.29835 (13)	0.46294 (17)	0.0377 (8)	
C24	0.4131 (5)	0.33440 (14)	0.40410 (18)	0.0402 (8)	
C25	0.5154 (5)	0.37647 (14)	0.41540 (18)	0.0409 (8)	
C26	0.6232 (5)	0.38829 (13)	0.48406 (18)	0.0397 (8)	
C27	0.7254 (5)	0.43149 (14)	0.4979 (2)	0.0455 (9)	
H27	0.7312	0.4551	0.4604	0.055*	
C28	0.8174 (5)	0.43826 (15)	0.5685 (2)	0.0521 (10)	
H28	0.8873	0.4664	0.5792	0.063*	
C29	0.8042 (5)	0.40256 (14)	0.6231 (2)	0.0469 (9)	
H29	0.8662	0.4078	0.6705	0.056*	
C30	0.6169 (4)	0.35370 (13)	0.54261 (18)	0.0388 (8)	
C31	0.5108 (4)	0.30925 (13)	0.53248 (18)	0.0374 (8)	
C32	0.3580 (5)	0.37973 (15)	0.30428 (19)	0.0446 (9)	
C33	0.2823 (5)	0.40102 (16)	0.23082 (19)	0.0482 (9)	
C34	0.1441 (6)	0.37953 (18)	0.1892 (2)	0.0624 (12)	
H34	0.0982	0.3507	0.2072	0.075*	
C35	0.0714 (7)	0.3997 (2)	0.1212 (3)	0.0758 (15)	
H35	−0.0223	0.3843	0.0938	0.091*	
C36	0.1369 (7)	0.4422 (2)	0.0939 (3)	0.0833 (16)	
H36	0.0882	0.4559	0.0481	0.100*	
C37	0.2733 (8)	0.4642 (3)	0.1343 (3)	0.101 (2)	
H37	0.3186	0.4931	0.1161	0.122*	
C38	0.3453 (7)	0.4437 (2)	0.2027 (3)	0.0862 (18)	
H38	0.4385	0.4593	0.2301	0.103*	
C39	0.4948 (5)	0.35307 (16)	0.8007 (2)	0.0493 (10)	
C40	0.4200 (5)	0.40138 (15)	0.82448 (19)	0.0451 (9)	
C41	0.4235 (6)	0.41146 (17)	0.8999 (2)	0.0554 (11)	
C42	0.3566 (6)	0.45676 (17)	0.9206 (2)	0.0618 (12)	
H42	0.3606	0.4638	0.9706	0.074*	
C43	0.2841 (6)	0.49147 (17)	0.8672 (2)	0.0591 (11)	
H43	0.2375	0.5216	0.8813	0.071*	
C44	0.2805 (5)	0.48158 (14)	0.7923 (2)	0.0462 (9)	
C45	0.3491 (5)	0.43707 (14)	0.77218 (19)	0.0456 (9)	
H45	0.3479	0.4308	0.7221	0.055*	
O1	0.5147 (4)	0.35041 (11)	0.73431 (14)	0.0549 (7)	
O2	0.5356 (4)	0.31814 (11)	0.84891 (15)	0.0637 (8)	
O3	0.4895 (5)	0.37738 (13)	0.95378 (15)	0.0761 (10)	
H3C	0.5247	0.3523	0.9347	0.114*	
O4	0.2865 (4)	0.52048 (11)	0.66450 (15)	0.0647 (8)	
O5	0.0217 (4)	0.50440 (12)	0.69531 (18)	0.0720 (9)	
O6	0.1886 (4)	0.57477 (10)	0.75266 (16)	0.0630 (8)	
OW1	0.6019 (4)	0.24205 (11)	0.75763 (15)	0.0530 (7)	
S1	0.18387 (13)	0.52366 (4)	0.72053 (5)	0.0505 (3)	
N1	0.8838 (4)	0.32410 (12)	0.77783 (16)	0.0455 (8)	
N2	0.8700 (4)	0.25209 (11)	0.67122 (15)	0.0430 (7)	
N3	1.3373 (4)	0.22601 (12)	0.92645 (16)	0.0470 (8)	
N4	1.3291 (4)	0.16684 (13)	0.83680 (17)	0.0434 (7)	
N5	0.5096 (4)	0.27879 (11)	0.59281 (15)	0.0414 (7)	
N6	0.7089 (4)	0.36156 (12)	0.61175 (15)	0.0417 (7)	
N7	0.3145 (4)	0.33662 (12)	0.33373 (15)	0.0423 (7)	
N8	0.4802 (4)	0.40487 (12)	0.35073 (15)	0.0447 (8)	
Zn	0.67652 (6)	0.304312 (19)	0.69016 (2)	0.04405 (16)	
H1WA	0.560 (6)	0.2603 (18)	0.788 (2)	0.085 (18)*	
H1WB	0.671 (4)	0.2195 (13)	0.776 (2)	0.058 (13)*	
H4B	1.342 (5)	0.1371 (16)	0.818 (2)	0.043 (11)*	
H8B	0.547 (6)	0.434 (2)	0.344 (3)	0.085 (17)*	

### Atomic displacement parameters (Å^2^)

**Table:**

	*U*^11^	*U*^22^	*U*^33^	*U*^12^	*U*^13^	*U*^23^
C1	0.064 (3)	0.042 (2)	0.044 (2)	0.0063 (19)	−0.0015 (19)	−0.0042 (17)
C2	0.079 (3)	0.048 (2)	0.041 (2)	−0.002 (2)	−0.002 (2)	−0.0106 (17)
C3	0.064 (3)	0.051 (2)	0.038 (2)	−0.003 (2)	−0.0128 (18)	−0.0074 (18)
C4	0.050 (2)	0.0380 (18)	0.0314 (17)	−0.0051 (17)	−0.0021 (15)	0.0011 (15)
C5	0.048 (2)	0.047 (2)	0.0324 (18)	−0.0059 (17)	−0.0038 (16)	0.0048 (16)
C6	0.045 (2)	0.044 (2)	0.0322 (17)	−0.0035 (17)	−0.0024 (15)	0.0022 (15)
C7	0.047 (2)	0.044 (2)	0.0267 (16)	−0.0041 (17)	0.0017 (15)	0.0040 (14)
C8	0.056 (2)	0.048 (2)	0.0356 (19)	0.0032 (18)	0.0089 (17)	0.0000 (16)
C9	0.066 (3)	0.056 (2)	0.0306 (18)	0.000 (2)	0.0017 (18)	−0.0087 (17)
C10	0.058 (2)	0.055 (2)	0.0294 (18)	0.001 (2)	−0.0065 (17)	−0.0026 (17)
C11	0.047 (2)	0.0413 (19)	0.0286 (17)	−0.0035 (17)	−0.0001 (15)	0.0037 (15)
C12	0.049 (2)	0.0410 (19)	0.0287 (17)	−0.0045 (17)	−0.0016 (15)	0.0019 (14)
C13	0.049 (2)	0.044 (2)	0.0316 (18)	−0.0045 (17)	−0.0055 (16)	0.0034 (15)
C14	0.045 (2)	0.045 (2)	0.040 (2)	−0.0043 (17)	0.0002 (16)	0.0065 (16)
C15	0.061 (3)	0.051 (2)	0.038 (2)	−0.0033 (19)	−0.0032 (18)	0.0024 (17)
C16	0.064 (3)	0.066 (3)	0.038 (2)	−0.004 (2)	−0.0071 (19)	0.0076 (19)
C17	0.059 (3)	0.062 (3)	0.051 (2)	0.003 (2)	−0.007 (2)	0.017 (2)
C18	0.062 (3)	0.047 (2)	0.063 (3)	0.001 (2)	0.003 (2)	0.009 (2)
C19	0.053 (2)	0.046 (2)	0.047 (2)	−0.0073 (18)	−0.0001 (18)	0.0042 (18)
C20	0.058 (2)	0.049 (2)	0.039 (2)	−0.0043 (19)	−0.0012 (18)	0.0097 (17)
C21	0.063 (3)	0.046 (2)	0.041 (2)	−0.0081 (19)	−0.0027 (18)	0.0044 (17)
C22	0.056 (2)	0.045 (2)	0.0347 (19)	−0.0025 (18)	−0.0071 (17)	0.0005 (16)
C23	0.044 (2)	0.0413 (19)	0.0252 (16)	0.0053 (16)	0.0013 (14)	−0.0024 (14)
C24	0.050 (2)	0.044 (2)	0.0238 (16)	0.0019 (17)	0.0010 (15)	−0.0011 (14)
C25	0.050 (2)	0.043 (2)	0.0270 (17)	0.0046 (17)	0.0020 (15)	0.0029 (15)
C26	0.048 (2)	0.0403 (19)	0.0274 (17)	0.0037 (16)	−0.0017 (15)	−0.0009 (14)
C27	0.058 (2)	0.041 (2)	0.0337 (19)	−0.0006 (18)	0.0008 (17)	0.0044 (15)
C28	0.061 (3)	0.045 (2)	0.045 (2)	−0.0053 (19)	−0.0036 (18)	−0.0003 (17)
C29	0.056 (2)	0.045 (2)	0.0335 (19)	−0.0041 (18)	−0.0068 (17)	−0.0026 (16)
C30	0.048 (2)	0.0364 (18)	0.0296 (17)	0.0071 (16)	0.0015 (15)	−0.0014 (14)
C31	0.043 (2)	0.0379 (18)	0.0291 (16)	0.0048 (16)	0.0020 (14)	−0.0001 (14)
C32	0.053 (2)	0.047 (2)	0.0319 (18)	0.0041 (18)	0.0035 (17)	0.0003 (16)
C33	0.059 (2)	0.056 (2)	0.0275 (17)	0.005 (2)	0.0025 (17)	0.0031 (16)
C34	0.075 (3)	0.064 (3)	0.040 (2)	−0.004 (2)	−0.010 (2)	0.006 (2)
C35	0.083 (3)	0.080 (3)	0.050 (3)	−0.006 (3)	−0.021 (2)	0.013 (2)
C36	0.092 (4)	0.102 (4)	0.046 (3)	0.009 (3)	−0.013 (3)	0.025 (3)
C37	0.112 (5)	0.120 (5)	0.061 (3)	−0.026 (4)	−0.012 (3)	0.047 (3)
C38	0.091 (4)	0.097 (4)	0.056 (3)	−0.029 (3)	−0.021 (3)	0.031 (3)
C39	0.055 (2)	0.055 (2)	0.035 (2)	0.0003 (19)	0.0029 (17)	0.0078 (18)
C40	0.053 (2)	0.050 (2)	0.0326 (19)	−0.0002 (18)	0.0077 (16)	0.0055 (16)
C41	0.068 (3)	0.064 (3)	0.032 (2)	0.000 (2)	0.0034 (18)	0.0131 (18)
C42	0.093 (3)	0.065 (3)	0.0281 (19)	0.008 (3)	0.013 (2)	0.0021 (19)
C43	0.082 (3)	0.056 (2)	0.040 (2)	0.001 (2)	0.015 (2)	−0.0049 (19)
C44	0.058 (2)	0.041 (2)	0.0368 (19)	−0.0056 (18)	0.0042 (17)	0.0010 (16)
C45	0.063 (2)	0.046 (2)	0.0279 (18)	−0.0028 (18)	0.0090 (17)	0.0049 (16)
O1	0.0694 (19)	0.0610 (17)	0.0317 (14)	0.0165 (14)	0.0037 (12)	0.0042 (12)
O2	0.088 (2)	0.0598 (17)	0.0427 (15)	0.0196 (16)	0.0106 (15)	0.0163 (13)
O3	0.115 (3)	0.079 (2)	0.0325 (14)	0.024 (2)	0.0107 (16)	0.0190 (14)
O4	0.102 (2)	0.0548 (17)	0.0377 (15)	−0.0002 (16)	0.0142 (15)	0.0096 (12)
O5	0.069 (2)	0.0657 (19)	0.070 (2)	−0.0097 (16)	−0.0132 (16)	0.0058 (16)
O6	0.090 (2)	0.0400 (15)	0.0534 (16)	0.0009 (15)	0.0012 (15)	−0.0008 (13)
OW1	0.070 (2)	0.0505 (17)	0.0360 (14)	0.0062 (15)	0.0031 (14)	0.0059 (13)
S1	0.0690 (7)	0.0400 (5)	0.0379 (5)	−0.0021 (5)	−0.0012 (5)	0.0026 (4)
N1	0.0531 (19)	0.0443 (17)	0.0342 (16)	0.0029 (15)	−0.0038 (14)	−0.0004 (13)
N2	0.0509 (18)	0.0456 (17)	0.0283 (15)	0.0036 (14)	−0.0026 (13)	0.0015 (13)
N3	0.0510 (19)	0.0470 (18)	0.0363 (16)	−0.0050 (15)	−0.0077 (14)	0.0020 (13)
N4	0.0508 (19)	0.0391 (18)	0.0361 (16)	0.0011 (15)	−0.0022 (14)	0.0026 (14)
N5	0.0493 (18)	0.0415 (16)	0.0288 (14)	−0.0026 (14)	−0.0035 (13)	0.0059 (12)
N6	0.0513 (18)	0.0471 (17)	0.0231 (14)	0.0014 (15)	−0.0014 (13)	−0.0002 (12)
N7	0.0512 (19)	0.0480 (18)	0.0246 (14)	0.0032 (14)	−0.0007 (13)	−0.0028 (13)
N8	0.059 (2)	0.0480 (18)	0.0236 (14)	0.0009 (16)	−0.0001 (14)	0.0014 (13)
Zn	0.0535 (3)	0.0465 (3)	0.0276 (2)	0.0027 (2)	−0.00348 (19)	0.0027 (2)

### Geometric parameters (Å, °)

**Table:**

C1—N1	1.324 (5)	C26—C30	1.407 (5)
C1—C2	1.384 (6)	C27—C28	1.380 (5)
C1—H1	0.9300	C27—H27	0.9300
C2—C3	1.360 (6)	C28—C29	1.382 (6)
C2—H2	0.9300	C28—H28	0.9300
C3—C4	1.400 (5)	C29—N6	1.318 (5)
C3—H3	0.9300	C29—H29	0.9300
C4—C12	1.410 (5)	C30—N6	1.362 (4)
C4—C5	1.426 (5)	C30—C31	1.442 (5)
C5—N3	1.371 (5)	C31—N5	1.360 (4)
C5—C6	1.393 (5)	C32—N7	1.322 (5)
C6—N4	1.359 (5)	C32—N8	1.359 (5)
C6—C7	1.432 (5)	C32—C33	1.478 (5)
C7—C11	1.397 (5)	C33—C38	1.368 (7)
C7—C8	1.410 (5)	C33—C34	1.369 (6)
C8—C9	1.361 (5)	C34—C35	1.377 (6)
C8—H8	0.9300	C34—H34	0.9300
C9—C10	1.389 (6)	C35—C36	1.367 (8)
C9—H9	0.9300	C35—H35	0.9300
C10—N2	1.332 (5)	C36—C37	1.354 (8)
C10—H10	0.9300	C36—H36	0.9300
C11—N2	1.354 (4)	C37—C38	1.386 (6)
C11—C12	1.468 (5)	C37—H37	0.9300
C12—N1	1.348 (5)	C38—H38	0.9300
C13—N3	1.324 (5)	C39—O1	1.261 (5)
C13—N4	1.378 (5)	C39—O2	1.265 (5)
C13—C14	1.460 (5)	C39—C40	1.501 (6)
C14—C19	1.386 (6)	C40—C45	1.379 (5)
C14—C15	1.404 (5)	C40—C41	1.401 (5)
C15—C16	1.371 (6)	C41—O3	1.359 (5)
C15—H15	0.9300	C41—C42	1.384 (6)
C16—C17	1.385 (7)	C42—C43	1.378 (6)
C16—H16	0.9300	C42—H42	0.9300
C17—C18	1.386 (6)	C43—C44	1.392 (5)
C17—H17	0.9300	C43—H43	0.9300
C18—C19	1.381 (6)	C44—C45	1.369 (6)
C18—H18	0.9300	C44—S1	1.776 (4)
C19—H19	0.9300	C45—H45	0.9300
C20—N5	1.315 (5)	O3—H3C	0.8200
C20—C21	1.392 (5)	O4—S1	1.461 (3)
C20—H20	0.9300	O5—S1	1.429 (3)
C21—C22	1.369 (5)	O6—S1	1.448 (3)
C21—H21	0.9300	OW1—H1WA	0.86 (2)
C22—C23	1.398 (5)	OW1—H1WB	0.840 (19)
C22—H22	0.9300	N4—H4B	0.86 (4)
C23—C31	1.417 (4)	N8—H8B	0.96 (5)
C23—C24	1.434 (5)	Zn—O1	2.080 (3)
C24—C25	1.374 (5)	Zn—OW1	2.196 (3)
C24—N7	1.388 (4)	Zn—N1	2.177 (3)
C25—N8	1.377 (4)	Zn—N2	2.184 (3)
C25—C26	1.429 (5)	Zn—N5	2.141 (3)
C26—C27	1.400 (5)	Zn—N6	2.120 (3)
			
N1—C1—C2	123.5 (4)	N5—C31—C30	117.4 (3)
N1—C1—H1	118.2	C23—C31—C30	121.5 (3)
C2—C1—H1	118.2	N7—C32—N8	112.4 (3)
C3—C2—C1	118.6 (4)	N7—C32—C33	125.8 (3)
C3—C2—H2	120.7	N8—C32—C33	121.7 (3)
C1—C2—H2	120.7	C38—C33—C34	117.5 (4)
C2—C3—C4	120.2 (3)	C38—C33—C32	121.2 (4)
C2—C3—H3	119.9	C34—C33—C32	121.3 (4)
C4—C3—H3	119.9	C33—C34—C35	121.6 (4)
C3—C4—C12	116.9 (3)	C33—C34—H34	119.2
C3—C4—C5	125.6 (3)	C35—C34—H34	119.2
C12—C4—C5	117.4 (3)	C36—C35—C34	120.0 (5)
N3—C5—C6	110.4 (3)	C36—C35—H35	120.0
N3—C5—C4	128.3 (3)	C34—C35—H35	120.0
C6—C5—C4	121.3 (3)	C37—C36—C35	119.5 (4)
N4—C6—C5	105.7 (3)	C37—C36—H36	120.3
N4—C6—C7	131.2 (3)	C35—C36—H36	120.3
C5—C6—C7	122.9 (3)	C36—C37—C38	120.1 (5)
C11—C7—C8	118.1 (3)	C36—C37—H37	119.9
C11—C7—C6	116.2 (3)	C38—C37—H37	119.9
C8—C7—C6	125.6 (4)	C33—C38—C37	121.3 (5)
C9—C8—C7	119.0 (4)	C33—C38—H38	119.3
C9—C8—H8	120.5	C37—C38—H38	119.3
C7—C8—H8	120.5	O1—C39—O2	124.6 (4)
C8—C9—C10	119.4 (4)	O1—C39—C40	117.8 (3)
C8—C9—H9	120.3	O2—C39—C40	117.6 (3)
C10—C9—H9	120.3	C45—C40—C41	118.9 (4)
N2—C10—C9	123.0 (3)	C45—C40—C39	120.2 (3)
N2—C10—H10	118.5	C41—C40—C39	120.8 (3)
C9—C10—H10	118.5	O3—C41—C42	118.6 (4)
N2—C11—C7	122.0 (3)	O3—C41—C40	121.6 (4)
N2—C11—C12	116.3 (3)	C42—C41—C40	119.8 (4)
C7—C11—C12	121.6 (3)	C43—C42—C41	120.2 (4)
N1—C12—C4	122.4 (3)	C43—C42—H42	119.9
N1—C12—C11	117.4 (3)	C41—C42—H42	119.9
C4—C12—C11	120.3 (3)	C42—C43—C44	120.1 (4)
N3—C13—N4	111.9 (3)	C42—C43—H43	119.9
N3—C13—C14	125.0 (3)	C44—C43—H43	119.9
N4—C13—C14	123.1 (3)	C45—C44—C43	119.4 (4)
C19—C14—C15	118.8 (3)	C45—C44—S1	118.1 (3)
C19—C14—C13	122.5 (3)	C43—C44—S1	122.5 (3)
C15—C14—C13	118.7 (4)	C44—C45—C40	121.5 (3)
C16—C15—C14	120.5 (4)	C44—C45—H45	119.2
C16—C15—H15	119.8	C40—C45—H45	119.2
C14—C15—H15	119.8	C39—O1—Zn	128.6 (3)
C15—C16—C17	120.3 (4)	C41—O3—H3C	109.5
C15—C16—H16	119.8	Zn—OW1—H1WA	99 (4)
C17—C16—H16	119.8	Zn—OW1—H1WB	119 (3)
C16—C17—C18	119.6 (4)	H1WA—OW1—H1WB	118 (5)
C16—C17—H17	120.2	O5—S1—O6	113.6 (2)
C18—C17—H17	120.2	O5—S1—O4	113.49 (19)
C19—C18—C17	120.3 (4)	O6—S1—O4	111.35 (19)
C19—C18—H18	119.9	O5—S1—C44	106.73 (18)
C17—C18—H18	119.9	O6—S1—C44	106.79 (17)
C18—C19—C14	120.5 (4)	O4—S1—C44	104.06 (19)
C18—C19—H19	119.7	C1—N1—C12	118.2 (3)
C14—C19—H19	119.7	C1—N1—Zn	127.7 (3)
N5—C20—C21	122.9 (4)	C12—N1—Zn	112.3 (2)
N5—C20—H20	118.5	C10—N2—C11	118.2 (3)
C21—C20—H20	118.5	C10—N2—Zn	127.9 (3)
C22—C21—C20	119.0 (4)	C11—N2—Zn	112.6 (2)
C22—C21—H21	120.5	C13—N3—C5	105.0 (3)
C20—C21—H21	120.5	C6—N4—C13	106.9 (3)
C21—C22—C23	119.8 (3)	C6—N4—H4B	127 (3)
C21—C22—H22	120.1	C13—N4—H4B	125 (3)
C23—C22—H22	120.1	C20—N5—C31	119.4 (3)
C22—C23—C31	117.8 (3)	C20—N5—Zn	127.5 (2)
C22—C23—C24	125.8 (3)	C31—N5—Zn	113.1 (2)
C31—C23—C24	116.4 (3)	C29—N6—C30	118.6 (3)
C25—C24—N7	110.1 (3)	C29—N6—Zn	127.6 (2)
C25—C24—C23	120.8 (3)	C30—N6—Zn	113.8 (2)
N7—C24—C23	128.9 (3)	C32—N7—C24	104.8 (3)
C24—C25—N8	105.9 (3)	C32—N8—C25	106.9 (3)
C24—C25—C26	124.5 (3)	C32—N8—H8B	133 (3)
N8—C25—C26	129.4 (3)	C25—N8—H8B	120 (3)
C27—C26—C30	118.9 (3)	O1—Zn—N6	92.25 (12)
C27—C26—C25	125.9 (3)	O1—Zn—N5	97.90 (12)
C30—C26—C25	115.1 (3)	N1—Zn—N2	76.13 (11)
C28—C27—C26	118.6 (3)	N6—Zn—N5	78.42 (11)
C28—C27—H27	120.7	O1—Zn—N1	93.32 (12)
C26—C27—H27	120.7	N6—Zn—N1	98.51 (12)
C27—C28—C29	119.0 (4)	N5—Zn—N1	168.46 (12)
C27—C28—H28	120.5	O1—Zn—N2	166.53 (10)
C29—C28—H28	120.5	N6—Zn—N2	97.52 (12)
N6—C29—C28	123.8 (3)	N5—Zn—N2	93.14 (11)
N6—C29—H29	118.1	O1—Zn—OW1	85.83 (12)
C28—C29—H29	118.1	N6—Zn—OW1	169.79 (11)
N6—C30—C26	121.1 (3)	N5—Zn—OW1	91.90 (11)
N6—C30—C31	117.3 (3)	N1—Zn—OW1	91.62 (11)
C26—C30—C31	121.6 (3)	N2—Zn—OW1	86.12 (12)
N5—C31—C23	121.1 (3)		

### Hydrogen-bond geometry (Å, °)

**Table:**

*D*—H···*A*	*D*—H	H···*A*	*D*···*A*	*D*—H···*A*
O3—H3C···O2	0.82	1.82	2.547 (4)	147
OW1—H1WA···O2	0.86 (2)	1.90 (3)	2.712 (4)	157 (5)
OW1—H1WB···N7^i^	0.84 (2)	2.05 (2)	2.877 (4)	170 (4)
N4—H4B···O6^ii^	0.86 (4)	2.05 (4)	2.883 (4)	162 (4)
N4—H4B···S1^ii^	0.86 (4)	3.02 (4)	3.854 (4)	163 (3)
N8—H8B···O4^iii^	0.96 (5)	1.85 (5)	2.794 (5)	167 (5)
N8—H8B···S1^iii^	0.96 (5)	2.94 (5)	3.793 (4)	148 (4)

Symmetry codes: (i) *x*+1/2, −*y*+1/2, *z*+1/2; (ii) −*x*+3/2, *y*−1/2, −*z*+3/2; (iii) −*x*+1, −*y*+1, −*z*+1.
